# Polymyositis and rhabdomyolysis caused by hepatocellular carcinoma - Case report and literature review

**DOI:** 10.1016/j.amsu.2021.102269

**Published:** 2021-04-04

**Authors:** Dávid Bárdos, Mária Judit Molnár, Ibolyka Dudás, Sebestyén Tuza, Attila Szijártó, Oszkár Hahn

**Affiliations:** a 1st Department of Surgery and Interventional Gastroenterology, Semmelweis University, Budapest, 1085, Budapest, Üllői út 78, Hungary; b Institute of Genomic Medicine and Rare Disorders, Semmelweis University, 1085, Budapest, Üllői út 78, Hungary; c Medical Imaging Centre, Semmelweis University, 1085, Budapest, Üllői út 78, Hungary; d 2^nd^ Department of Pathology, Semmelweis University, 1085, Budapest, Üllői út 93, Hungary

**Keywords:** Rhabdomyolysis, Polymyositis, Hepatocellular carcinoma, Paraneoplastic syndromes, Case report

## Abstract

**Introduction:**

Rhabdomyolysis is a syndrome characterized by a rapid necrosis of muscle fibers and the release of muscle-derived metabolic products into the circulatory system. A rare cause of rhabdomyolysis is paraneoplastic polymyositis.

**Case presentation:**

A 67-year-old man was diagnosed with paraneoplastic polymyositis and rhabdomyolysis caused by hepatocellular carcinoma (HCC). Intravenous steroid was used as a symptomatic therapy for rhabdomyolysis, and the tumour was removed by left hemihepatectomy to treat the underlying cause. After muscle strength gradually improved, steroid therapy was discontinued. The patient was reoperated multiple times due to bleeding and bile leakage. Following the operations, his overall state and muscle strength further improved. Despite that, the patient's condition worsened again, and eventually, he died of candida albicans pneumonia and sepsis.

**Discussion:**

HCC is an extremely rare cause of paraneoplastic polymyositis and rhabdomyolysis. Treatment is challenging, as none of the few available case reports record long term survival and less than half of the reports record muscle strength improvement. In our case, the patient was treated with systemic steroid therapy and resection of the tumour. The patient's muscle strength temporarily improved, but subsequently, the patient died.

**Conclusion:**

Our case confirms the importance of a definitive treatment of HCC, as we achieved a significant improvement in muscle strength by removing the tumour. On the other hand, our paper highlights the dangers of double-sided steroid therapy, which, combined with the essential, effective treatment of rhabdomyolysis, may have contributed to the development of postoperative complications and candida sepsis leading to death.

## Highlights

• Hepatocellular carcinoma is an extremely rare cause of paraneoplastic polymyositis.
• Definitive treatment of paraneoplastic polymyositis is surgical removal of the tumour.
• Steroid therapy is an essential treatment of polymyositis and rhabdomyolysis.
• Steroid therapy also increases the risk of postoperative complications.
• Steroid therapy, combined with surgical treatment may lead to increased postoperative risk.

## 1 Introduction

Rhabdomyolysis is a syndrome characterized by the rapid necrosis of muscle fibers and the release of muscle-derived metabolic products into the circulatory system. The classic triad of symptoms is muscle weakness, muscle pain and tea-brown urine. The gold standard examination is serum creatinine kinase (CK) test, which shows at least 5-fold, but usually more than 10-fold elevation from the normal value [1,2]. A common, dangerous consequence of rhabdomyolysis is renal failure caused by metabolic products accumulating in the bloodstream. Rhabdomyolysis can be caused by trauma, heavy physical exertion, drugs, inherited neuromuscular diseases, or other systemic causes [3]. Systemic causes include paraneoplastic polymyositis as well. According to a review, the most common causes of paraneoplastic polymyositis are lung cancer and non-Hodgkin lymphoma [4]. However, another study found lung, ovary, breast, colorectal and nasopharyngeal tumours the most likely to develop paraneoplastic polymyositis and dermatomyositis [5]. In this paper, we present a rare case of rhabdomyolysis developed due to paraneoplastic polymyositis caused by hepatocellular carcinoma.

There are only seven documented cases of paraneoplastic polymyositis associated with hepatocellular carcinoma in the international literature, out of which only four cases resulted in rhabdomyolysis.

In the first case of the rhabdomyolysis group, polymyositis was treated with intravenous steroid therapy and hemodialysis due to the acute renal failure. The patient's renal function improved, but his muscle strength did not. Prior to his planned chemotherapy treatment, the patient died of tumour rupture [6]. In another case, the patient's muscle symptoms regressed after steroid therapy and liver resection, but the patient died of pulmonary embolism shortly after the surgery [7]. In a third case, a patient with Hepatitis B-induced cirrhosis was treated for liver failure when first liver cancer, and then rhabdomyolysis was detected. He received fluid therapy to prevent kidney failure and was not treated with steroids. The patient died of liver failure on day 30 of admission [8]. In a fourth case, a hepatitis B positive cirrhotic patient with bilobary located irresectable tumour was diagnosed with rhabdomyolysis. The patient received transarterial chemoembolization (TACE) and steroid therapy. Serum CK level was reduced as a result, but muscle strength was not improved. The patient died of pneumonia 6 months later [9].

In three other cases, rhabdomyolysis was not diagnosed in addition to HCC-induced paraneoplastic polymyositis. In one case, the patient was treated with steroids, and the patient refused TACE treatment for his irresectable tumour. His muscle strength did not improve, and he died of liver failure within two months [10]. In another case, the patient was treated with steroids and metothrexate and died within 3 months [11]. Finally, a further case described HCC-induced polymyositis in which the patient's symptoms and elevated CK improved after liver resection without pharmacotherapy [12].

Five out of the seven patients were male, the patients’ age varied between 37 and 72 years.

All but one of the patients were first examined for musculoskeletal symptoms, and one for cirrhosis. Among the patients, tumour-free liver parenchyma abnormalities were found in two cases, both patients had hepatitis B virus-positive (HBV +) cirrhosis. Liver parenchyma was not described in the other cases. Rhabdomyolysis caused kidney failure in only one case, which improved after successful treatment. Serum creatinine kinase levels were elevated in all available cases at the time of diagnosis and were decreased in all reported cases, mostly to normal level. In six of the seven cases, polymyositis was treated with steroids, supplemented in one case with metothrexate and in one case with nonsteroidal anti-inflammatory drugs. Surgery was performed in two cases, TACE treatment was applied in two other cases, and the tumour was not treated in the remaining cases. Muscle strength improved in three cases, in one case in the rhabdomyolysis group (the patient who was treated with surgery) and in two patients in the group without rhabdomyolysis.

The findings of the case reports are summarized in Table 1.

**Table 1:** HCC-induced polymyositis and rhabdomyolysis – literature review.

Author	Kim [6]	Kishore [7]	Gidaagaya [8]	Thanapirom [9]	Tekaya [10]	Iba-Ba [11]	Hasegawa [12]
**Age**	55	50	73	56	72	37	70
**Sex**	man	woman	woman	man	man	man	man
**Muscle symptoms**	progressive proximal muscle weakness of the lower limbs	proximally more severe progressing symmetrical quadriparesis and neck weakness	lower extremity weakness	proximal 4 limb and neck weakness	proximally more severe 4 limb weakness	dysphagia and arthromyalgia of the limbs	proximal weakness of limb muscles and neck muscles
**Kidney function**	oliguria, acute renal failure solved by hemodialysis	normal	normal	normal	not mentioned	not mentioned	not mentioned
**First symptoms**	weakness	weakness for 6 weeks	cirrhosis	weakness and fever	weakness	arthromyalgia	weakness for 1 year
**Tumour-free liver parenchyma**	normal	normal	HBV + cirrhosis	HBV + cirrhosis	normal	normal	normal
**Initial CK**	40,000	1972	3554	17963	1710	~1500	1443
**Rhabdomyolysis**	yes	yes	yes	yes	no	no	no
**Polymyositis therapy**	1 mg/kg methyl- prednisolone for 5 weeks	1 mg/kg methyl- prednisolone for 5 weeks	supportive to prevent renal failure	1 mg/kg prednisolone	corticosteroid and non- steroid	bolus 750 mg/d for 3 days then 50 m/week methotrexate, 50 mg corticosteroid	none
**Tumour therapy**	chemotherapy planned but patient died	left hepatolobectomy	none	TACE	TACE planned but patient denied	best supportive care	segment 7–8 resection following TAE
**Renal function after treatment**	improved	normal	normal	normal	not mentioned	not mentioned	not mentioned
**Muscle weakness after treatment**	no significant improvement	significant improvement	not mentioned	persisted	persisted	improved	improved
**Post-treatment CK**	unknown	normal	1831	normal	unknown	normal	normal
**Follow-up**	died in tumour rupture	died in pulmonary embolism	died in hepatic failure	died in pneumonia	died in hepatic failure	died in septic shock	tumour-free, weakness resolved
**Survival from the onset of symptoms**	about 60 days	about 6.5 mo	1 mo	6.5 mo	5 mo	not mentioned	alive after 16 mo
**Survival from the diagnosis**	54 days	about 4 mo	1 mo	5 mo	2 mo	3 mo	alive after 1 mo

HCC-related polymyositis was described in 5 additional cases in which polymyositis was considered to be a drug side effect [[13], [14], [15], [16], [17]].

## 2 Presentation of the case

This case is presented in line with the SCARE 2020 criteria [18].

A 67-year-old caucasian man was referred by his family physician to our surgery department with progressive fatigue, muscle weakness, and a weight loss of 11 kg that had started six weeks before. Earlier, he had had no symptoms and his muscle strength had been normal. Within six weeks, he developed swallowing difficulties and severe, predominantly proximal muscle weakness in all four limbs. He was unable to stand up and walk without assistance.

He had a history of hypertension, diabetes mellitus type 2, central retinal vein occlusion, vitrectomy due to vitreous hemorrhage, and open repair of scrotal hernia.

Laboratory test confirmed extremely high CK values (CK 15,409 IU/L), elevated C reactive protein (CRP 36 mg/L), and macroalbuminuria. Renal function was normal (creatinine 46 μM/L, GFR [glomerular filtration rate]> 90 mL/min/1.73 m^2^). He had no history of trauma, heavy physical exertion, or drug/toxic abuse. The patient had not received any new medication in the period leading up to the examination. Immunological studies had ruled out autoimmune origin, as anti-DNA (anti-Deoxyribonucleic Acid), antinuclear antibody, atypical ANCA (Anti-Neutrophil Cytoplasmic Antibody), ANCA Myeloperoxidase EIA/CIA, proteinase 3-ANCA at EIA/CIA, anti-Jo, ANTI Mi, SSA and SSB (Sjogren Syndrome A and B), CCP (cyclic citrullinated peptide) tests were negative.

**Fig. 1:**
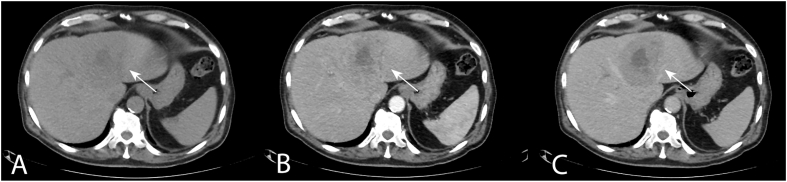
Abdominal CT scan, axial slices. A tumour mass visible in segment 4 invading a small portion of segment 2 of the liver (white arrow). Radiological features are typical for HCC: early contrast enhancement and early wash-out with a non-enhancing central scar.

Further investigations were performed due to a suspicion of paraneoplastic syndrome. Abdominal ultrasonography found an 8 × 7x7 cm foreign tissue in the liver, the AFP was increased. Aspiration cytology confirmed HCC. Abdominal CT scan confirmed a 10 cm solitary mass in the liver, mainly located in segment 4 invading a smaller part of segment 2 and distracting the middle and left hepatic vein with the typical radiographic features of HCC: early arterial contrast enhancement and washout on portal venous phase with a hypodens nonenhancing necrotic central scar. No sign of metastasis was seen neither in the hilar lymph nodes, nor far away, and no other abnormality was visible on CT scan (Fig. 1). Core biopsy confirmed well-differentiated HCC. Examination of hepatitis A, B, C and E ended with a negative result. No abnormality was found on the chest X-ray.

**Fig. 2:**
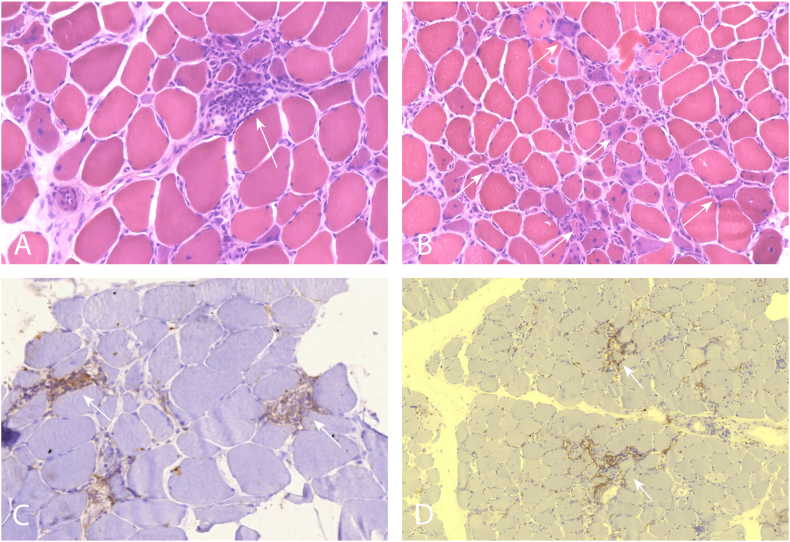
Histological examination of quadriceps muscle biopsy. **A, B** Hematoxylin-eosin staining, 350X and 260× magnification. The diameter of the muscle fibers are varying. Mononuclear infiltration and necrotizing muscle fibers are visible. White arrows pointing towards muscle fibers with signs of inflammation and necrosis. **C** CD4 immunostaining, 350× magnification. CD4 positive cell infiltration is visible. White arrow points to areas densely infiltrated by CD4 positive cells. **D** CD8 immunostaining, 200× magnification. CD8 positive cell infiltration is visible. White arrow points to areas densely infiltrated by CD8 positive cells.

Muscle biopsy taken from the quadriceps diagnosed inflammatory necrotizing myopathy with diffuse atrophic fibers, a great proportion of necrotic fibers and mononuclear infiltration with variable intensity in multiple locations. Immunohistochemical staining showed CD4, CD8 cells and CD68 positive fibers confirming inflammatory activity. On the surface of muscle fibers, increased MHC-1 expression was visible. Typical skin lesions and histologic changes referring to dermatomyositis were absent (Fig. 2).

Based on the findings above, we diagnosed HCC-induced paraneoplastic polymyositis and consequent rhabdomyolysis in the background of the patient's symptoms.

To control progressive muscle weakness, we considered liver resection as the first and most important step to treat HCC. Chemotherapy and transarterial chemoembolization are non-curative techniques and, according to previous case reports, we believed they would not solve the paraneoplastic symptoms. Tumour size was too large for radiofrequency ablation (RFA). Thus, we decided to surgically remove the tumour as the first and most important step to treat paraneoplastic inflammatory myopathy and HCC. Based on imaging studies, the tumour appeared removable by left hepatolobectomy. Therefore, with the patient's informed consent, despite of the increased risk due to his poor overall condition, surgery was planned. Preoperatively, we supplemented nutrition with a high protein oral and parenteral formula (Protifar and Kabiven). We also administered steroid therapy (methylprednisolone 125 mg iv.) 4 days before surgery.

Subsequently, open left hepatolobectomy was performed by a hepatic surgery expert in the hepatopancreatobiliary surgical center of the surgery department of our university hospital.

Pathological examination confirmed necrotizing Grade 3 pT2 hepatocellular carcinoma with vascular invasion. The lesion reached the resection margin in the line of the vena cava (vascular R1). Tumour-free liver tissue showed typical features of portal inflammation and fibrosis.

After surgery, the patient was admitted to the intensive care unit as planned. On the fourth postoperative day, the sedation, mechanical ventilation, and circulatory support could be discontinued.

On postoperative day 2, steroid therapy was reduced to 80 mg/day methylprednisolone, and the dose was gradually reduced and eventually abandoned after five weeks. With intensive physiotherapy, bed biking and mobilization, the patient's muscle strength gradually improved in all four limbs and the trunk. The patient was able to stand up and walk with assistance six weeks after surgery. Following the introduction of steroid therapy and surgery, serum CK levels decreased abruptly (CK 3339 IU/L on postoperative day 1) and then gradually decreased to normal range (CK 95 IU/L) over a six-week period and remained in the normal range.

On postoperative day 9, bleeding was observed through the abdominal drain, which urged an acute reoperation. During the surgery, we found that the bleeding of the resection surface of the liver had been caused by the mechanical friction of the drain. The operation was performed by an expert hepatic surgeon. Following reoperation, intensive therapeutic care was continued. After reoperation, the patient got pneumonia and sepsis, which improved after the administration of targeted antibiotic treatment. The patient's overall condition gradually improved.

Through his remaining fistula opening at the site of his drain, we repeatedly experienced minor bleeding that did not cause circulatory fluctuations. We reoperated the patient again. During the second reoperation, hematoma evacuation and bleeding control was performed. The hematoma recurred once again and was treated with percutaneous drainage. Subsequently, bile leakage and intraabdominal fluid collection was found and the patient was reoperated again. The remaining stump of the left bile duct was necrotic and leaking, hence left hepatic duct suture and bile drainage was performed with the insertion of an open biliary T-tube (Kehr's tube). All reoperations were performed by an expert hepatic surgeon. After a temporary improvement, his condition gradually deteriorated, and he developed sepsis again.

No complication was observed on the abdominal CT scan. On the chest CT-scan, bilateral pneumonia was seen. Tracheal aspirate culture was positive for Candida albicans, which is known to be an opportunistic pathogen. When the patient was conscious, he could squeeze his hand tightly and elevate his lower limbs when prompted.

**Fig. 3:**
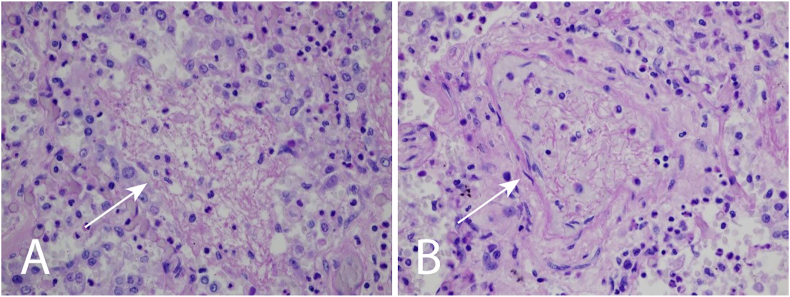
Post mortem lung tissue sample, periodic acid-Schiff (PAS) reaction, 600X magnification. **A** Fungal filaments appearing in the alveolar space (white arrow) surrounded by inflammatory cells indicate candida pneumonia. **B** Intravascular fungal filaments (white arrow) indicate candida sepsis.

With an increasing need for circulatory support, the patient died in sepsis and multiorgan failure on the 81st postoperative day. The autopsy and post-mortem lung tissue samples confirmed bilateral candida pneumonia and candida sepsis as the cause of death, there was no abnormality in the abdomen (Fig. 3).

## 3 Discussion

In this paper, we described a rare case of polymyositis and rhabdomyolysis associated with hepatocellular carcinoma. The patient was treated with corticosteroids, supplementary nutrition, and the tumour was resected. After the resection of the tumour, the clinical signs and laboratory parameters of the polymyositis regressed and the patient's muscle strength gradually improved. The corticosteroid therapy was relatively quickly discontinued. The patient underwent multiple reoperations to control bleeding and bile leakage. Despite of an initial improvement, the patient developed candida pneumonia and candida sepsis and eventually died.

Paraneoplastic polymyositis and rhabdomyolysis are extremely rare complications of HCC. There are only seven reports of HCC-induced polymyositis in the literature [[6], [7], [8], [9], [10], [11], [12]], with only four cases when polymyositis was associated with rhabdomyolysis [[6], [7], [8], [9]]. In six cases, polymyositis was treated with steroid therapy, in one case steroid and metothrexate were used [11], and in one case only intravenous fluid therapy was applied to prevent renal failure [8]. Elimination of the underlying cause, i.e. surgery, was performed in two cases [7,12], no surgery was performed in the other cases due to the irresectability of the tumour or the poor general condition of the patient. The muscle strength of both two operated patients improved. In contrast, among the patients who did not undergo surgery, muscle strength improved in only half of the cases, and there was no improvement in the other half. No long-term survival was reported, the longest documented survival was 6.5 months from the onset of symptoms and 5 months from diagnosis [9]. Causes of death included liver failure in two cases [8,10], tumour rupture [6], pneumonia [9], and pulmonary embolism in one case [7], respectively. Cause of death was not documented in the remaining cases.

Based on the previously documented cases and our own experience, HCC-induced polymyositis and rhabdomyolysis is a difficult-to-treat disease with poor prognosis. Therapy should cover the paraneoplastic immunologic process of rhabdomyolysis, renal failure (if presented), and the cause of the disease, HCC at the same time. This puts clinicians in a difficult position, as poor general condition and steroid therapy increases the risk of postoperative complications after liver resection. However, treatment effectiveness of less invasive ablation or embolization is questionable.

## 4 Conclusion

Our observation underlines the importance of a definitive surgical therapy of HCC, since we achieved a significant improvement in muscle strength by removing the tumour. On the other hand, our paper highlights the dangers of double-sided steroid therapy, which, in addition to the essential, effective treatment of rhabdomyolysis, may have contributed to the development of postoperative complications and candida sepsis leading to death.

## Provenance and peer review

Not commissioned, externally peer reviewed.

## Sources of funding

The authors received no financial support for the research, authorship, and/or publication of this article.

## Ethical approval

The institutional review board approved the article.

## Consent

Written informed consent was obtained from the patient for publication of this case report and accompanying images. A copy of the written consent is available for review by the Editor-in-Chief of this journal on request.

## Author contribution

Dr. Dávid Bárdos – data collection, writing the paper, assisting at surgeries

Prof. Dr. Mária Judit Molnár – study concept and design, interpreting muscle biopsy, treating rhabdomyolysis

Dr. Ibolyka Dudás – data collection, interpreting radiological findings

Dr. Sebestyén Tuza – data collection, interpreting pathological findings

Prof. Dr. Attila Szijártó – data interpretation, reoperations

Dr. Oszkár Hahn – study concept and design, data interpretation, hepatic resection, reoperations.

## Trial registry number

N/a.

## Guarantor

Dr. Dávid Bárdos.

Dr. Oszkár Hahn.

## Declaration of competing interest

None.

## Appendix A Supplementary data

SCARE checklist SCARE checklist
